# First Survey of SNPs in *TMEM154*, *TLR9*, *MYD88* and *CCR5* Genes in Sheep Reared in Italy and Their Association with Resistance to SRLVs Infection

**DOI:** 10.3390/v13071290

**Published:** 2021-07-01

**Authors:** Chiara Arcangeli, Daniele Lucarelli, Martina Torricelli, Carla Sebastiani, Marcella Ciullo, Claudia Pellegrini, Andrea Felici, Silva Costarelli, Monica Giammarioli, Francesco Feliziani, Fabrizio Passamonti, Massimo Biagetti

**Affiliations:** 1 Istituto Zooprofilattico Sperimentale dell’Umbria e delle Marche-Togo Rosati (IZSUM), Via Salvemini 1, 06126 Perugia, Italy or chiara.arcangeli1@studenti.unipg.it (C.A.); d.lucarelli@izsum.it (D.L.); m.ciullo@izsum.it (M.C.); c.pellegrini@izsum.it (C.P.); a.felici@izsum.it (A.F.); s.costarelli@izsum.it (S.C.); m.giammarioli@izsum.it (M.G.); f.feliziani@izsum.it (F.F.); m.biagetti@izsum.it (M.B.); 2 Dipartimento di Medicina Veterinaria, Università degli Studi di Perugia, Via San Costanzo 4, 06126 Perugia, Italy; fabrizio.passamonti@unipg.it

**Keywords:** small ruminant lentiviruses (SRLVs), transmembrane protein 154 (*TMEM154*), toll-like receptor 9 (*TLR9*), myeloid differentiation primary response 88 (*MYD88*), chemokine (C-C motif) receptor 5 (*CCR5*), genetic resistance, relative risk

## Abstract

*Maedi-visna* virus (MVV) and caprine arthritis encephalitis virus (CAEV), referred to as small ruminant lentiviruses (SRLVs), belong to the genus *Lentivirus* of the Retroviridae family. SRLVs infect both sheep and goats, causing significant economic losses and animal welfare damage. Recent findings suggest an association between serological status and allelic variants of different genes such as *TMEM154*, *TLR9*, *MYD88* and *CCR5*. The aim of this work was to investigate the role of specific polymorphisms of these genes in SRLVs infection in some sheep flocks in Italy. In addition to those already known, novel variants in the *TMEM154* (P7H, I74V, I105V) gene were detected in this study. The risk of infection was determined finding an association between the serological status and polymorphisms P7H, E35K, N70I, I74V, I105V of *TMEM154*, R447Q, A462S and G520R in *TLR9* gene, H176H* and K190K* in *MYD88* genes, while no statistical association was observed for the 4-bp deletion of the *CCR5* gene. Since no vaccines or treatments have been developed, a genetically based approach could be an innovative strategy to prevent and to control SRLVs infection. Our findings are an important starting point in order to define the genetic resistance profile towards SRLVs infection.

## 1. Introduction

*Maedi*-*visna* virus (MVV) and caprine arthritis encephalitis virus (CAEV) belong to the genus *Lentivirus* of Retroviridae family. For a long time, they were considered specifically related to sheep and goats respectively, both in terms of host tropism and clinical features.

However, at a later time, molecular epidemiological studies showed that the two viruses represent a spectrum of variants able to infect both sheep and goats in the field. Therefore, this viruses in this are referred to as Small Ruminant Lentiviruses (SRLVs) [1]. A high viral mutation rate, in association with the selective pressure can determine evolution of different viruses, altering both pathogenesis and viral tropism. Indeed, SRLVs tropism, based on the host–virus interaction, takes place at different levels: cellular, organ/tissue or host. In vivo, the monocyte, the main SRLVs target cell, once infected, behaves as a “Trojan horse” capable of spreading the virus in the body, maintaining the infection latent until it differentiates into macrophage, activating transcription of viral genes [2]. After an initial viremic phase, seroconversion occurs after several months [3] and the classical clinical symptoms, that are strain-dependent, might show up afterwards and sometimes even years [4]. Generally, SRLVs cause pneumonia, weakness, mastitis, polyarthritis, encephalomyelitis and progressive paralysis affecting animal productions and welfare [5]. The lungs, joints, udders and nervous system are the main target organs. Horizontal transmission occurs through direct contact between animals and by respiratory secretions [6]. Vertical transmission is mainly based on the lactogenic route, in particular through the ingestion of colostrum and milk from infected dams [7].

SRLVs have a worldwide distribution with the exception of Iceland, New Zealand and Australia, which are currently the only MVV-free countries, but not CAEV-free. Due to the lack of a specific legislation dealing with official control programs, many countries such as the Netherlands, Finland, Germany, Switzerland, France, Spain and Italy, implemented voluntary eradication plans of questionable effectiveness. Epidemiological data available on SRLVs show an increasing seroprevalence in flocks, especially in Europe, which worry farmers for the huge economic damages due to trade restrictions, reduced milk production compromising lamb growth, increased mortality of newborns and premature culling of infected animals [5]. The World Organization for Animal Health (OIE) has included SRLVs in the list of notifiable terrestrial and aquatic animal diseases, due to the high socio-economic impact on international trade of animals and their products [8]. 

The most relevant surveillance tools for SRLVs infection are based on serological tests. Currently, no vaccine has been developed and the conventional management strategies are questionable and not economically feasible, particularly on high-productivity farms. Therefore, considering the limitations of traditional strategies for the containment of SRLVs infection, a genetically-based approach could represent the most suitable mean to effectively control the disease [9,10]. Various studies focused on the identification of Single Nucleotide Polymorphisms (SNPs) in target genes, involved in SRLVs resistance, like *TMEM154*, *CCR5*, *MHC* genes, *ZNF389*, *TLRs*, *MYD88* and *APOBEC3* [11,12,13]. In particular, a genome wide association study (GWAS) conducted in North America revealed a correlation between variants of transmembrane protein 154 (*TMEM154*) gene and resistance/susceptibility to SRLVs infection [14]. *TMEM154* gene is located on chromosome 17 and the sequence is composed by seven exons. The amino acid (aa) chain includes a signal peptide (1-30 aa), an extracellular domain (31-82 aa), a transmembrane region (83-106 aa) and a cytoplasmatic region (107-191 aa) [14]. *TMEM154* gene encodes for a precursor protein of 191 amino acids that mature in a protein of 161 amino acids, but its biological function is still unknown. Some studies showed that the amino acid substitution glutamate tolysine (E/K) in position 35 (rs408593969) can modulate the predisposition to the infection, in particular the KK genotype is associated to a decrease in susceptibility together with low serological levels [9,15,16,17,18,19]. However, other authors reported that the KK genotype is not fully protective depending on several possible factors as viral dose, route of infection, type and number of SRLVs strains involved, comorbidities, other environmental and genetic factors [14,19].

Due to the possible role played by *TMEM154* gene in SRLVs susceptibility in sheep, further polymorphisms were identified leading to the definition of 12 different haplotypes, among which the most common are haplotypes 1, 2, and 3 [15,18]. 

Phylogenetic studies defined the haplotype 3 of the *TMEM154* gene, characterized by a glutamate (E) in position 35 and an asparagine (N) in position 70 (rs427737740), as the ancestral one. Haplotypes 1 and 2 differ from haplotype 3 for the presence of K in position 35 and of I in position 70, respectively. Animals homozygous for haplotype 2 and 3 do not show differences between each other in serological status, suggesting that the mutation in position 70 (N70I) does not play a significant role in infection resistance [14]. Furthermore, animals carrying at least one copy of either haplotype 2 or 3 seem to be almost three times more susceptible than animals with two copies of haplotype 1 [11]. This association has also been confirmed by other studies in some USA, German, Iranian and Turkish flocks [9,18,19]. Haplotype 4 is characterized by a single nucleotide deletion (R4AΔ53) in position 4 (rs594936094) that determines a frame-shift mutation and a premature stop codon in position 54 predicted to cause the loss of the protein function [14]. Animals with two copies of haplotype 4 did not show clinical symptoms despite being bred together with infected animals, but further analysis would be necessary to confirm this observation [15]. 

In addition, another target investigated in this field is the protein encoded by the ovine chemokine (C-C motif) receptor 5 (*CCR5*) gene, located on chromosome 19 and formed by two exons [19,20]. The scientific interest paid to the potential role of *CCR5* in SRLVs resistance derived from studies concerning Human Immune-deficiency Virus (HIV) infection where individuals carrying a 32-bp deletion (*CCR5*-Δ32) in the coding region showed high genetic resistance to HIV [21]. A role of *CCR5*-Δ32 in genetic resistance has been observed also for hepatitis B virus [22]. However, other studies showed its involvement in disease severity and genetic susceptibility to respiratory syncytial and West Nile viruses and in increased mortality to influenza A virus in CCR5-deficient mice [23,24,25]. 

Since SRLVs are macrophage-tropic lentiviruses likewise HIV, a number of flocks was analyzed to assess if the deletion in the *CCR5* gene could exist, eventually contributing to the resistance to SRLVs infection [11,20,26]. Actually, a *CCR5* four base pairs deletion, (*CCR5*-Δ4-rs119102753) was associated to a lower proviral load of SRLVs together with a lower *CCR5* gene expression [20].

Additionally, two other genes seem to be involved in genetic resistance to SRLVs, namely Toll-Like Receptor 9 (*TLR9*) and its mediator Myeloid differentiation primary response 88 (*MYD88*) [27]. TLRs variants have been found to be involved in resistance in many infectious diseases including porcine salmonellosis, ovine brucellosis and bovine paratuberculosis [28].

TLRs are a family of transmembrane receptors expressed on plasma and endosomal membranes of multiple cellular types (e.g., epithelial cells, macrophages, natural killer cells and dendritic cells). They are able to recognize the pathogen-associated molecular patterns (PAMPs), including viral components, then triggering the innate immune response mechanisms through the activation of NF-kB-mediated production of pro-inflammatory cytokines [12,13]. In particular, TLR9 localization in intracellular compartments is critical for the recognition of CpG motifs in bacterial and viral nucleic acids. Upon stimulation of cells, TLR9 redistributes from the endoplasmic reticulum (ER) to lysosomes, triggering a signaling cascade that involves the recruitment of the MyD88 adaptor molecule [29].

Polymorphisms in *TLR9* and *MYD88* genes potentially associated to SRLVs transmission were investigated by Sarafidou [27]. Different polymorphisms were identified in the *TLR9* gene sequence, among which the wild type (*wt*) G520R polymorphism seems to be protective against infection. On the other hand, regarding *MYD88* gene, only two synonymous SNPs were identified [27], but further studies are needed to assess and clarify its specific role in resistance to SRLVs infection.

To the best of our knowledge, no studies have been conducted in Italy to identify gene variants involved in the genetic resistance to SRLVs in sheep. The purpose of this study was to carry out for the first time a survey to investigate the already known and de novo SNPs in *TMEM154*, *CCR5*, *TLR9* and *MYD88* target genes in some flocks reared in Italy, in order to identify variants that could confer resistance to SRLVs infection. The preliminary results obtained in this research suggest a possible correlation between some genotypes of *TMEM154*, *TLR9*, and *MYD88* and resistance to SRLVs infection in sheep flocks in Italy, but they need to be confirmed in a larger sheep population. In any case, prior to the future adoption and implementation of marker-assisted selection (MAS), it will be of paramount importance to define any potential effect of these selected favorable alleles on other traits, mainly on the productive ones. This approach could offer the possibility to breed resistant animals to prevent the disease occurrence. In this way, it could be possible to reduce veterinary costs, limiting the treatments and the elimination of infected animals meanwhile increasing production and profitability.

## 2. Materials and Methods

### 2.1. Sample Collection and DNA Extraction

A total of 493 sheep (*Ovis aries*) from 91 Italian sheep flocks, in particular reared in Central and Southern Italy, were sampled to evaluate serological SRLVs status. The sampled age-categories were represented by over three-year-old animals. No ethical approval was required, in compliance with the European Directive 2010/63/UE and the Italian Regulation D. Lgs n. 26/2014: for the purpose of the study, aliquots of blood samples, taken during obligatory routine animal sanitary controls by authorized veterinarians, were used. After serum separation, residual blood clots were stored at −20°C until analysis. Prior to DNA extraction, 200 mg of each blood clot was digested with 500 μL Tissue Lysis Buffer and 80 μL Proteinase K (both from Roche Life Science, Mannheim, Germany) at 65 °C for 1h. Genomic DNA extraction was performed using High Pure PCR Template Preparation Kit (Roche Life Science), following the manufacturer’s instructions.

### 2.2. PCR Amplification and Sequencing of TMEM154, TLR9 and MYD88 Genes

PCR amplifications of all target genes were performed in a final volume of 50 µL containing 60–100 ng of genomic DNA using a Mastercycler Ep Gradient S (Eppendorf AG, Hamburg, Germany). Oligonucleotides were purchased by Thermo Fisher Scientific, Waltham, MA, USA (Table 1).

Specific primers for exons 1/2 of *TMEM154* gene were selected from Heaton [14] and the corresponding PCR amplification mixes contained respectively: 1.5 X GoTaq^®^ Flexi Buffer, 1/1.3 mM MgCl_2_, 1.5 /2.5 U GoTaq^®^ G2 Flexi DNA Polymerase (Promega Corporation, Madison, WI, USA), 200 µM dNTPs (GE Healthcare, Buckinghamshire, England), 0.4 µM of each primer and 5% DMSO only for exon 2 (Sigma-Aldrich, Saint Louis, MO, USA). Both PCR protocols were carried out with the following thermal cycling profile: an initial denaturation step at 95 °C for 5 min followed by 35 cycles at 95 °C for 1 min, 52 °C for 30 s, 72 °C for 1 min and a final extension step at 72 °C for 7 min.

*TLR9* gene and *MYD88* gene were amplified according to Sarafidou [27]. PCR reactions were set up as follows: 1 X GoTaq^®^ Flexi Buffer, 1.3 mM MgCl_2_, 1 U of GoTaq^®^ G2 Flexi DNA Polymerase (Promega Corporation), 150 µM dNTPs (GE Healthcare) and 0.12 µM of each primer (Thermo Fisher Scientific). The amplification protocol was performed with the following thermal cycling profile: an initial denaturation step at 95 °C for 5 min followed by 35 cycles at 95 °C for 30 s, 62 °C for 40 s, 72 °C for 40 s and a final extension step at 72 °C for 7 min.

PCR products of all target genes were subjected to 2% agarose gel electrophoresis containing Midori Green Advanced DNA Stain (Nippon Genetics Europe GmbH, Düren, Germany) and were purified with QIAquick^®^ PCR Purification Kit (Qiagen, Hilden, Germany), according to the manufacturer’s instructions. The quantity and quality of the PCR products were assessed photometrically using a Biophotometer (Eppendorf). Sequencing reactions were carried out in both directions with the same primers used for PCR amplifications, using BrilliantDye^TM^ Terminator Cycle Sequencing Kit v3.1 (NimaGen BV, Nijmegen, The Netherlands) in accordance with the manufacturer’s instructions. Sequencing reactions were run in a 3500 Genetic Analyzer (Thermo Fisher Scientific). All sequences, in FASTA format, were aligned to *Ovis aries* genes *TMEM154* (GenBank Accession Number HM355886.3), *TLR9* (GenBank Accession Number NM_001011555.1) and *MYD88* (GenBank Accession Number NM_001166183.1) and analyzed with BioEdit v7.2.5 software [30], using ClustalW algorithm. In addition, electropherograms were checked at each investigated mutation point to locate heterozygous peaks.

### 2.3. Allelic Discrimination Assay by Real-Time PCR (qPCR) of CCR5 Gene

The presence of the deletion in the promoter region of the ovine *CCR5* gene was assessed by an allelic discrimination assay through qPCR using primers and probes set according to White [20]. Particularly, the reaction mix was prepared in a final volume of 25 μL containing: 1× TaqMan^TM^ Genotyping Master Mix (Thermo Fisher Scientific), 0.6 μM and 0.3 μM of forward (5′-GCAAGTGGGTCAGAATCTCTCA-3′) and reverse (5′-CACCCAACTACCCAAATGGTTAGAA-3′) primers (Thermo Fisher Scientific), respectively, 0.15 μM of both *CCR5*-wt probe (5′-VIC-CTCATGAATGTTCTTCT-3′MGBNFQ) and *CCR5*-mutated probe (5′-6FAM-ATGCTCATGTTCTTCT-3′MGBNFQ) (Diatech Pharmacogenetics, Jesi, Italy). qPCR amplification was performed using a 7500 Fast Real-Time PCR system (Thermo Fisher Scientific) using the following thermal cycling conditions: initial steps at 50 °C for 20 s and at 95 °C for 20 s followed by 40 cycles at 95 °C for 3 s and 57 °C for 30 s. The results were analyzed using the 7500 Software v2.3 (Thermo Fisher Scientific). 

### 2.4. Serological Test

Serological tests were performed in duplicate on serum samples using the commercial ELISA assay (ID Screen^®^ MVV/CAEV Indirect Screening test–IDvet, Grabels, France) that detects gp135 and p25 antibodies, following the manufacturer’s instructions. Raw data were acquired by the Sunrise^TM^ Basic Tecan spectrometer equipped with a Magellan^TM^ reader control and data processing software v7.1 (Tecan Group Ltd., Männedorf, Switzerland). The cut-off value was defined based on the corrected optical density (OD) ratio between sample and positive control (S/P) at a wavelength of 450 nm:

(1) $$S/P\left(\%\right)=\frac{\mathrm{mean}\;\mathrm{OD}\;\mathrm{value}\;\mathrm{of}\;\mathrm{samples}-\;\mathrm{mean}\;\mathrm{OD}\;\mathrm{value}\;\mathrm{of}\;\mathrm{negative}\;\mathrm{controls}\;}{\mathrm{mean}\;\mathrm{OD}\;\mathrm{value}\;\mathrm{of}\;\mathrm{positive}\;\mathrm{controls}-\;\mathrm{mean}\;\mathrm{OD}\;\mathrm{value}\;\mathrm{of}\;\mathrm{negative}\;\mathrm{controls}}\times 100$$

According to the manufacturer’s instructions, samples are considered SRLVs negative with an S/P value ≤50% and SRLVs positive with an S/P value ≥60%. Doubtful results, given with a value in the range of 50–60%, were excluded from further analysis.

### 2.5. Statistical Analysis

Haplotype frequencies were calculated dividing the number of copies of each haplotype by the total of haplotypes. 

The square of the correlation coefficient (r^2^) was used to evaluate the linkage disequilibrium (LD) using the R Studio software [31]. The significance level of *p*-value was set at 0.05. 

The most probable haplotypes of the *TMEM154* and *TLR9* genes were calculated using PHASE v2.1 software [32] that performs the best reconstruction of haplotypes in a population, based on Bayesian inference so on probabilistic events.

The correlations between genotypes and resistance/susceptibility to SRLVs infection status, determined by antibodies detection, were performed evaluating the relative risk (RR) with 95% confidence interval according to Altman [33] using the following equation:

(2) $$\mathrm{RR}=\frac{a/\left(a+b\right)}{c/\left(c+d\right)}$$

where a is the number of serologically-positive individuals carrying the risk factor, b is the number of serologically negative individuals carrying the risk factor, c is the number of serologically positive individuals carrying no risk factor and d is the number of serologically negative individuals carrying no risk factor. 

*p*-value was calculated according to Sheskin [34] computing a standard normal deviate (z-value) as ln(RR)/SE{ln(RR)}, where SE is the Standard Error: the *p*-value is the area of the normal distribution that falls outside ±z. MedCalc Software [35] was used for calculations. 

A high RR value is related to predisposition to the disease, in particular the risk and protective alleles were considered statistically significant with a *p*-value < 0.05.

## 3. Results

### 3.1. TMEM154, TLR9, MYD88 and CCR5 Genotyping

Sequence analysis of *TMEM154* exons 1 and 2 revealed seven known missense polymorphic sites (R4AΔ53, T25I, E35K, G38R_rs1088921014, T44M_rs420489630, N70I, E82YΔ82) and three novel missense SNPs not previously detected, in particular, one polymorphism in the signal peptide (P7H), one in the extracellular domain (I74V) and one in the transmembrane region (I105V). The new mutation I74V, instead of I74F found by other authors, has to be highlighted [14,18,36].

Genetic variations that generate amino acid substitutions gave rise to 15 different and unambiguous haplotypes that included 100% of the observed ones as a result of the presence of only one polymorphic site in heterozygosity. Specifically, we identified six already known (1, 2, 3, 4, 6, 16) and nine novel haplotypes (17–25) reported in Table S1.

Haplotypes 1 and 3 were the most common in the studied flocks, showing frequencies of 0.483 and 0.298 respectively, followed by haplotypes 21 (0.095), while the remaining haplotypes were present with frequencies less than 0.05. 

Haplotypes 4 and 25 derived from a frame-shift deletion at codon position 4 (A4Δ53) differing each other for an arginine at amino acid position 44. Similarly, haplotype 6 originated from a deletion at position 82 (E82YΔ82). Both frame-shift deletions are predicted to determine premature termination of the amino acid chain thus abolishing protein function.

Haplotypes 17 and 18 differed from haplotypes 1 and 3 respectively, for a histidine amino acid residue at position 7, whereas haplotype 20 was the only characterized by a methionine at amino acid position 44. In addition, regarding the above-mentioned novel I74V and I105V variants, haplotypes 21 and 24 presented both of them, while haplotypes 19 and 22 contained only the valine at position 74.

Sequence analysis of *TLR9* gene revealed three known missense mutations (R447Q, A462S, G520R). The detected polymorphisms (Table S2) pointed out the presence of unambiguous haplotypes that comprised 94% of those observed because they contained only one polymorphic site. Specifically, we identified five already known (3, 4, 5, 6, 11) and two novel (12, 13) haplotypes obtained from the combination of the previously mentioned polymorphic sites. Haplotype 11 is the most common with a frequency of 0.747 followed by haplotype 4 and 5 with frequencies of 0.126 and 0.117, respectively. Haplotypes 12 and 13 differ from haplotype 3 for an alanine at position 462 and for an arginine in position 447, respectively. 

Regarding *MYD88*, two synonymous nucleotide substitutions (T528C and A570C), not determining an amino acid change in the protein sequence, were detected. For better understanding, the mutated amino acids were indicated in the text with the symbol of asterisk (H176H* and K190K*). The two polymorphisms are in complete LD (r^2^ = 1.00, *p*-value < 0.001) and only two different haplotypes were identified: *MYD88_1* for *wt* and *MYD88_2* for the mutated form with a frequency of 0.194 and 0.806, respectively (Table S2).

Finally, qPCR analysis of *CCR5* polymorphism revealed a frequency of the deleted allele (*CCR5*-Δ4) of 0.299 and a frequency of the *wt* allele of 0.701.

### 3.2. Relative Risk of TMEM154, TLR9, MYD88 and CCR5 Polymorphisms

Seroprevalence of SRLVs in the investigated population was 41.8% (*n* = 206). The relative risk has been calculated on genotypes containing the alleles considered predisposing to the infection compared to those carrying the protective ones, as reported in Table 2. The risk genotypes of the *TMEM154* are HH and PH (RR = 1.64) at position 7, EE and EK (RR = 1.24) at position 35, NI and II (RR = 1.89) at position 70, II (RR = 2.61) at position 74, II (RR = 3.06) at position 105. On the other hand, the protective *TMEM154* genotypes are PP (RR = 0.61) at position 7, KK (RR = 0.80) at position 35, NN (RR = 0.53) at position 70, VV (RR = 0.38) at position 74 and VV (RR = 0.33) at position 105. Anyway, results relative to E35K genotypes were not significant even if they narrowly missed the significant threshold (Table 2).

Regarding *TLR9*, the risk genotypes are QQ (RR = 2.40) at position 447, AA and AS (RR = 1.96) at position 462, RR and GR (RR = 1.72) at position 520, while the protective ones are RR and RQ (RR = 0.42) at position 447, SS (RR = 0.51) at position 462 and GG (RR = 0.58) at position 520.

Finally, for *MYD88* the risk genotypes are HH and HH* (RR = 1.33) at position 176, KK and KK* (RR = 1.33) at position 190 and the protective ones are H*H* (RR = 0.75) at position 176 and K*K* (RR = 0.75) at position 190.

As regards the *CCR5* gene, no statistically significant association was found between the deleted genotype and the serological status.

## 4. Discussion

The investigation of genes involved in resistance/susceptibility to SRLVs infection has been conducted by several authors in order to identify SNPs as markers to be used as an alternative approach to control the infection, ensuring animal welfare and reducing the significant economic losses in infected flocks [5]. However, in Italy, few data on the frequencies of genetic variants are available and only regarding goat flocks in Northern and Southern Italy [26,37,38].

In this work, the frequencies of the known and novel risk/protective genotypes of the *TMEM154*, *TLR9*, *MYD88* and *CCR5* genes were assessed. These genes are involved in the innate immunity response pathway to pathogens, with the exception of *TMEM154* which role is still to be clarified. The only information available about *TMEM154* is its high level of expression in human cells of the monocyte lineage, that represents also the SRLVs cellular target [14]. The variability in the sequences of these candidate genes could interfere at different levels with the progression of the infection.

Concerning *TMEM154,* in this study we evaluated the relationship between gene polymorphisms and the deriving haplotypes as detailed in Table S1. Particularly, we found a total of 15 haplotypes of the *TMEM154* gene, nine of which have not been detected yet in other sheep populations. Haplotypes 1 and 3 were the most common in our flocks, showing a frequency of 0.483 and 0.298, respectively, while haplotype 2 had a low frequency (0.013). Haplotypes 2 and 3, both carrying the ancestral E35 residue but differing each other at position 70 for the codified amino acid, respectively an isoleucine or an asparagine, are predicted to confer high susceptibility to SRLVs infection. On the other hand, haplotype 1, containing K35 compared to the ancestral one, has been associated to resistance [14]. In our work, as can be inferred from RR calculation (Table 2), animals carrying the EE and EK genotypes seem to show a greater predisposition to SRLVs infection (RR = 1.24) than animals carrying the KK genotype (RR = 0.80); however, this result was not significant even if it approached the significant threshold (*p*-value = 0.055). Other authors similarly reported that the KK genotype is not fully protective depending on several factors concerning the host, the virus and the environment [14,19].

Regarding the novel SNPs at position 7, one or two copies of the mutated amino acid histidine were associated to risk of infection (RR = 1.64) as well as NI and II genotypes at position 70 (RR = 1.89). The H7 variant was only found in haplotypes 17 and 18 that were present at very low frequencies (Table S1) in the population object of this study. An aspect to highlight is that haplotype 18, containing other risk alleles (E35, I74 and I105), was mainly detected in seropositive animals (67%). This observation needs further investigations and in-depth analysis to be applied to a larger population.

Interestingly, as above mentioned, in our population we found the novel 74V variant instead of the already known 74F [15,18,36] and the new polymorphic site at position 105 coding for a valine rather than an isoleucine. Both were frequently observed in seronegative animals and were associated with a low susceptibility as can be deduced from RR calculation (Table 2). In fact, animals carrying both genotypes II at positions 74 and 105 had about 3-fold increased risk of infection respect to animals carrying the VV genotypes at the same positions. The combination of I74V and I105V mutated variants gave rise to the new haplotypes 21 and 24 found with a frequency of 0.095 and 0.002, respectively.

An aspect to highlight is that, in the investigated sheep, haplotype 21 has been detected in homozygosity in 46 animals, 39 of which were seronegative (85%). In addition, it was present in heterozygosity with haplotype 24 in two animals both seronegative.

These preliminary data suggest that this novel genetic profile may be considered a good candidate marker, despite the presence of the risk E allele at position 35. This finding request further investigation to better understand the involvement of these variants in the host’s genetic resistance to SRLVs.

Furthermore, haplotype 4, carrying a frame-shift deletion at codon position 4 (A4Δ53), has been associated to resistance. Indeed, animals with two copies of haplotype 4 did not show clinical symptoms despite being bred together with infected animals [15] but further analysis would be necessary to better define its role. On the other hand, Clawson [18] reported the ability of a specific SRLVs subgroup to infect animals homozygous for haplotype 4.

In the investigated population haplotype 4 was detected with a low frequency (0.011) as well as the novel haplotype 25 (0.007), differing each other only for an arginine at amino acid position 44. Unfortunately, since the low frequencies observed, a statistically significant association with the serological status was not obtained.

Regarding *CCR5* gene, a lower frequency of the *CCR5-*Δ4 allele (0.299) with respect to the *wt* allele (0.701) was found and no statistical correlation with disease resistance or susceptibility was observed in our survey similarly to what observed by Molaee [19].

For *TLR9* gene, data analysis revealed three polymorphic sites and their combination determined a total of seven haplotypes (Table S2), two of which were novel (haplotype 12, 13). We found that haplotype 11 was the most common with a frequency of 0.747 followed by haplotypes 4 and 5 with frequencies of 0.126 and 0.117, respectively. As results from relative risk calculation (Table 2), animals carrying genotype QQ at position 447 or one/two copies of A462 or R520 variants showed a risk of infection about 2-fold greater than those without.

The latter finding is partially in accordance with Sarafidou [27], that reported the association with seropositivity only for homozygous RR animals. Furthermore, the results showed that haplotype 4 (Table S2) contained protective variants, in particular R at 447 position, G at 520 position and R at 462 position, suggesting a potential resistant role in disease progression.

Relatively to the *MYD88* gene, the already-known polymorphic sites (H176H* and K190K*) [27], were observed also in the studied ovine population. In particular the mutated homozygous genotypes H*H* and K*K* were associated for the first time, to the best of our knowledge, to protection towards SRLVs infection as demonstrated in Table 2. Indeed, the association between *MYD88* genotypes and susceptibility/resistance was significant, although the only two involved polymorphisms, found in complete LD (r^2^ = 1.00; *p*-value < 0.001), gave rise to conservative mutations to be not under evaluated. In this context, the involvement of silent mutations in mRNA splicing and stability, enzymatic activity, protein folding, gene expression and function has been already highlighted [39,40,41]. This phenomenon, known as codon usage, implies that, among synonymous codons, the codon mostly used is that for which the corresponding tRNA is more abundant in cells. It was demonstrated that impairment of this mechanism due to genetic mutations could play an important role in the susceptibility or in the pathogenesis of some human diseases such as dilated cardiomyopathy, Treacher–Collins syndrome, cystic fibrosis, and hemophilia B [42].

On this basis, we can therefore hypothesize that the mutated haplotype of *MYD88* could exert a protective role against SRLVs infection due to the phenomenon of codon usage. However, further studies have to be conducted in other sheep populations to confirm this finding.

Our survey focused on a preliminary association between significant single polymorphisms found in the investigated population and relative risk of SRLVs infection.

Considering that the combination among different SNPs could alter this trend, a future important goal could be to conduct, with a higher sample number, a resistance/susceptibility association study to the haplotypes yet defined in this work. The application of molecular methods could be an alternative tool in SRLVs infection control.

Implementation of MAS schemes is a strategy that allows the increase of resistance traits to infectious diseases ensuring at the same time animal welfare and veterinary costs reduction in affected herds.

## 5. Conclusions

To date, few studies have been conducted in Italy to identify protective genetic variants and investigations have been carried out mainly in goat herds raised in Northern and Southern Italy. Significant associations of *TMEM154, TLR9* and *MYD88* variants to SRLVs resistance/susceptibility were found in the Italian sheep population studied in this report. To confirm the preliminary findings of our work, further investigations, also on other sheep populations, would be necessary with the final aim to adopt and to implement MAS programs by crossbreeding animals carrying these protective variants, after assessing possible undesirable effects on other traits. In fact, the exclusion of susceptible animals predisposed to SRLVs infection would limit both the development of clinical symptoms and the spread of the virus. Furthermore, this could also reduce trade restrictions and economic losses resulting from the disease. Thus, a genetically-based approach could be an innovative strategy to control SRLVs infection, especially in high productive sheep flocks.

## Figures and Tables

**Table 1:** Primer sequences of target genes.

Target gene	Primers Sequence	PCR Product Size	References
*TMEM154*	For 5′-GCGAGGCGTGCTAACTG-3′ Rev 5′-GCCCATTAAAGCCGGT-3′	589 bp	[14]
	For 5′-GAGGGTAAGTTTCAGATCATTG-3′ Rev 5′-TTATGTAGCTGCTTCACTTAAA-3′	554 bp	
*TLR9*	For 5′-TTCGTGGACCTGTCGGAC-3′ Rev 5′-CTGGCTGTTGTAGCTGAG-3′	414 bp	[27]
*MYD88*	For 5′-AGCCTGAGTATTTTGATGCC-3′ Rev 5′-ACCTGGAGAGAGGCTGAGC-3′	441 bp	

For: forward primer; Rev: reverse primer; bp: base pair.

**Table 2:** Significant relative risk of infection for *TMEM154*, *TLR9* and *MYD88* genotypes.

Gene	Polymorphism	Genotype	RR	95% CI	*p*-Value
*TMEM154*	P7H	HH, PH *vs.* PP	1.64	1.26–2.14	0.0002
		PP *vs.* HH, PH	0.61	0.47–0.79	
	E35K	EE, EK *vs.* KK	1.24	0.99–1.56	0.055 ^#^
		KK *vs.* EE, EK	0.80	0.64–1.01	
	I70N	NI, II *vs.* NN	1.89	1.31–2.73	0.0006
		NN *vs.* NI, II	0.53	0.37–0.76	
	I74V	II *vs.* VV	2.61	1.47–4.64	0.0010
		VV *vs.* II	0.38	0.22–0.68	
	I105V	II *vs.* VV	3.06	1.47–4.64	0.0020
		VV *vs.* II	0.31	0.16–0.65	
*TLR9*	R447Q	QQ *vs.* RQ, RR	2.40	2.16–2.67	<0.0001
		RR, RQ *vs.* QQ	0.42	0.37–0.46	
	A462S	AA, AS *vs.* SS	1.96	1.22–3.14	0.0053
		SS *vs.* AA, AS	0.51	0.32–0.82	
	G520R	RR, GR *vs.* GG	1.72	1.14–2.59	0.0101
		GG *vs.* RR, GR	0.58	0.39–0.88	
*MYD88*	H176H*	HH, HH* *vs.* H*H*	1.33	1.09–1.64	0.0060
		H*H* *vs.* HH, HH*	0.75	0.61–0.92	
	K190K*	KK, KK* *vs.* K*K*	1.33	1.09–1.64	0.0060
		K*K* *vs.* KK, KK*	0.75	0.61–0.92	

* Amino acid from mutated codon; P: proline, H: histidine, E: glutamic acid, K: lysine, I: isoleucine, N: asparagine, V: valine, R: arginine, Q: glutamine, A: alanine, S: serine, G: glycine; 95% CI: 95% confidence interval; ^#^ value close to the significant threshold.

## Data Availability

Data supporting reported results are available in this article and in the supplementary materials.

## Supplementary Materials

The following are available online at https://www.mdpi.com/article/10.3390/v13071290/s1, Table S1: *TMEM154* haplotype frequencies detected in the studied ovine population, Table S2: *TLR9* and *MYD88* haplotype frequencies detected in the studied ovine population.
