# Supplementary material for: First Survey of SNPs in TMEM154, TLR9, MYD88 and CCR5 Genes in Sheep Reared in Italy and Their Association with Resistance to SRLVs Infection
Source: Viruses. 2021 Jul 1;13(7):1290. doi: 10.3390/v13071290 (PMC8310241; doi:10.3390/v13071290)
Supplement: Supplementary file 1 [file viruses-13-01290-s001.zip › viruses-1262400-supplementary.pdf]

Table S1. *TMEM154* haplotype frequencies detected in the studied ovine population.

TMEM154	aa position									Haplotype
Haplotype	4	7	25	35	38	44	70	74	82	105	frequencies
**1**	R	P	T	K	G	T	N	I	E	I	0.483
**2**	R	P	T	E	G	T	I	I	E	I	0.013
**3**	R	P	T	E	G	T	N	I	E	I	0.298
**4**	A	P[a]	P[a]	N[a]	E[a]	W[a]	NA	NA	NA	NA	0.011
**6**	R	P	I	E	G	T	N	I	Y	NA	0.002
**16**	R	P	T	E	R	T	N	I	E	I	0.004
**17**	R	H	T	K	G	T	N	I	E	I	0.003
**18**	R	H	T	E	G	T	N	I	E	I	0.037
**19**	R	P	T	K	G	T	N	V	E	I	0.002
**20**	R	P	T	E	G	M	N	I	E	I	0.010
**21**	R	P	T	E	G	T	N	V	E	V	0.095
**22**	R	P	T	E	G	T	N	V	E	I	0.018
**23**	R	P	I	K	G	T	N	I	E	I	0.014
**24**	R	P	I	E	G	T	N	V	E	V	0.002
**25**	A	P[a]	P[a]	N[a]	E[a]	R[a]	NA	NA	NA	NA	0.007

aa: amino acid**;** R: arginine, P: proline, H: histidine, A: alanine, T: threonine, E: glutamic acid, K: lysine, G: glycine, M: methionine, W: tryptophan, N: asparagine, I: isoleucine, V: valine, Y: tyrosine. [a] result of frame-shift mutation. NA: not applicable due to premature stop codon.

Table S2. *TLR9* and *MYD88* haplotype frequencies detected in the studied ovine population.

TLR9 Haplotype	aa position			Haplotype
	447	462	520	frequencies
**3**	Q	S	R	0.002
**4**	R	S	G	0.126
**5** [a]	R	A	G	0.117
**6** [a]	Q	S	G	0.002
**11**[a]	R	A	R	0.747
**12**	Q	A	R	0.005
**13**	R	S	R	0.005
***MYD88* Haplotype**	**176**	**190**		
**1**	H	K		0.194
**2**	H*	K*		0.806

aa: amino acid; S: serine, G: glycine, R: arginine, Q: glutamine, A: alanine, H: histidine, K: lysine; [a]Haplotype nomenclature according to Sarafidou et al., 2013 ;* aminoacid from mutated codon
